# Identification, expression analysis of quinoa betalain biosynthesis genes and their role in seed germination and cold stress

**DOI:** 10.1080/15592324.2023.2250891

**Published:** 2023-08-24

**Authors:** Yang Feng, Xingzhu Yan, Fenggen Guo, Shiyi Wang, Zhengjie Liu, Wenhong Long

**Affiliations:** a College of Agronomy and Biotechnology, Yunnan Agricultural University, Kunming, China; b College of Horticulture and Landscape, Yunnan Agricultural University, Kunming, China

**Keywords:** *Chenopodium quinoa*, betalain biosynthesis, gene identification, gene cloning, expression analysis

## Abstract

Betalains provide *Chenopodium quinoa* bright color, and the key enzyme genes for betalain biosynthesis include *CYP76AD*, *DODA*, and *GTs*. In this study, 59 *CqCYP76AD*, *CqDODA* and *CqGTs* genes in quinoa were identified and characterized by gene structural characteristics, phylogenetic relationships and gene expression patterns. The *CqCYP76AD* genes were divided into ɑ, β and γ types, *CqDODA* into ɑ and β types, and *CqGTs* into *CqcDOPA5GT*, *CqB5GT* and *CqB6GT* types according to phylogenetic relationships. The analysis of co-linearity identified eight pairs of duplicated genes which were subjected to purifying selection during evolution. *CqCYP76AD* and *CqDODA*, as well as *CqcDOPA5GT* and *CqB5GT* may have been evolutionarily linked in genetic inheritance, based on gene location and gene structure study. The tissue expression specificity of *CqCYP76AD*, *CqDODA*, and *CqGTs* genes in response to seed germination and cold stress was studied by RNA-Seq data. The genes *CqCYP76AD*, *CqDODA*, and *CqGTs* were involved in betalain biosynthesis and cold stress. *CqCYP76AD*, *CqDODA*, *CqcDOPA5GT* and *CqB5GT* gene sequences were consistent in the eight quinoa samples and showed significant variations in expression. In contrast, the inconsistency between changes in gene expression and betalain accumulation indicates that other factors may influence betalain biosynthesis in quinoa. This study offers the theoretical basis for the roles of the *CqCYP76AD*, *CqDODA*, and *CqGTs* genes in betalain biosynthesis and cold stress in quinoa, as well as a guide for the full utilization of betalains in quinoa plants.

## 1. Introduction

Betalains are nitrogenous water-soluble pigments which biosynthesize in the cytoplasm and stored in the vacuole, providing the plant vibrant red, purple, yellow and white color.^1–3^ At present, all known betalains belong to the category of quaternary ammonium alkaloids, divided into the amino acid-based type of betaxanthins and the glycosylated type of betacyanins, with the absorbance spectra of 532~550 nm and 457~485 nm for betacyanins and betaxanthins.^2,4,5^ Betalains are mainly found in Caryophyllales plants and higher fungi, where they fulfill similar functions to anthocyanins.^6–9^ Betalains provide plants their vibrant colors and attract pollinators, it are also associated to plant stress resistance.^6,10–12^ The *Suaeda salsa* may grow on soils with 3% salt, which may be associated to betalain accumulation in their tissues.^13^ Cactaceae can maintain plant cell osmotic pressure through the synthesis and degradation of betaxanthin, which is one of the ways Cactaceae adapt to drought situations.^6,14^ In fact, betalain is an important natural pigment that can be used as a food supplement, cosmetic colorant, and has antioxidant, antitumor, liver protection and other health care medical values.^15–19^ Research has shown that betalains can scavenge hypochlorite by acting on peroxidases in human bone marrow.^20^ Prickly Pear fruit eating may increase the body’s levels of antioxidants, and prickly pear extract possesses potent antibacterial and antioxidant abilities in vivo.^21^ As a result, eating foods that contain betalain may assist in relieving the body of diseases induced by external stress.

**Figure 1.**
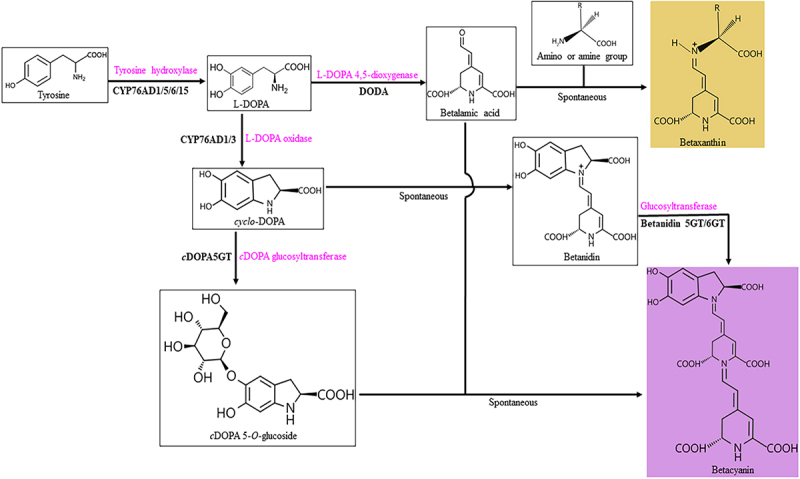
Betalain biosynthesis pathway (adapted from Alfonso Timoneda, et al 2019). The red colours indicate the type of enzyme required in betalain biosynthesis.

Betalain biosynthesis involves three types of genes CYP76AD, DODA, and GTs (Figure 1). Tyrosine is hydroxylated by cytochrome P450 (CYP) to L-3,4-dihydroxyphenylalanine (L-DOPA). L-DOPA can be catalyzed by L-DOPA oxidase to create 5,6-dihydroxyindoline-2-carboxylic acid (cyclo-DOPA) or by L-DOPA 4,5-dioxygenase (DODA) to create betalamic acid.^22,23^ Betalamic acid is a crucial phase in the biosynthesis of betalains.^24,25^ Cytochrome P450 is a key player in the betalains biosynthesis and has been isolated from *Beta vulgaris*, *Mirabilis jalapa*, and *S. salsa*.^26–28^ L- DOPA accumulation is necessary for the biosynthesis of betalamic acid, which inhibits L- DOPA from being further oxidized to dopaquinone.^29^ L-DOPA-4,5 dioxygenase is a ferritin that lacks hemoglobin that catalyzes the conversion of L-DOPA to seco-DOPA. By intramolecular condensation with the amino group, the seco-DOPA forms the backbone structure of the betalains and the chromogenic group betalamic acid.^29,30^ Betalamic acid incorporates spontaneously with amino acids or amines to produce betaxanthins, with only two enzymes involved in the process, cytochrome P450 and L-DOPA-4,5 dioxygenase.^31,32^ Betalamic acid can also react spontaneously with *cyclo*-DOPA to form betanidin, which are then glycosylation by betanidin 5-O-glucosyltransferase or betanidin 6-O-glucosyltransferase to form betacyanins, or *cyclo*-DOPA is first converted to cDOPA 5-O-glucoside by *c*DOPA glucosyltransferase, which then react spontaneously with betalamic acid to form betacyanins.^33–35^ Three groups of five enzymes play a role in the change from tyrosine to betalains, including cytochrome P450, L-DOPA-4,5 dioxygenase, and glucosyltransferase. The production of betacyanins includes three glucosyltransferases, and further research is needed to determine how betanidin 5-O-glucosyltransferase and betanidin 6-O-glucosyltransferase operate in the same plant. The genes for betanidin 5-O-glucosyltransferase and betanidin 6-O-glucosyltransferase have only been cloned from *Dorotheanthus bellidiformis*, and their sequences of amino acids are only 15% identical, and the two genes belong to different glucosyltransferase gene groups.^36^ Highly pure B5GT and B6GT enzymes derived from *D. bellidiformis* suspension cultures catalyze the transfer of glucose from UDP-glucose to the hydroxyl groups of betalains, flavonols, anthocyanins, and flavonoids, but differ in their affinity for the hydroxyl groups of the respective receptor compounds.^36^ The only difference between B5GT and B6GT enzymes is the location of the hydroxyl groups of glucose transferred to the receptor compounds, but chromatographic, electrophoretic, and kinetic properties suggest that the enzymatic activities of B5GT and B6GT are based on the presence of a single enzyme.^37^ The main function of B6GT in betaxanthins biosynthesis is to catalyze the transfer of glucose to the hydroxyl group of betalain sapogenins, but the position of the acceptor compound’s hydroxyl group and other biological functions of B6GT need to be investigated further.^36,37^

Quinoa (*Chenopodium quinoa* Willd.) is a member of the group caryophyllales, and study has revealed that it is the betalain, not anthocyanin, that gives quinoa its vivid color.^38,39^ Quinoa has become a popular crop in barren, saline, and drought locations due to its inherent drought and salt tolerance, and these benefits are attributed to its high amount of betalains.^6,10,12,40^ Some research has been conducted on quinoa betalains extraction and isolation, as well as structure identification.^38,39^ However, further study on the molecular mechanisms of its biosynthesis and regulation is needed. Quinoa’s stems, leaves, flowers and fruits are vibrantly colored. Quinoa is categorized into four types based on the color of the seeds: red, yellow, white, and black quinoa.^41^ Quinoa stems and leaves from several genetic resources might have the same color. Three red quinoas, three white quinoas, and two black quinoas were used as experimental materials in this research. In quinoa, 59 betalain biosynthesis genes were identified and their gene structure, conserved motif composition, evolutionary relationships and cis-acting elements were thoroughly studied. Meanwhile, the tissue-specific expression of quinoa betalain biosynthesis genes, as well as their expression response during cold stress and seed germination, was studied using quinoa cold stress RNA-seq data, expression data from different quinoa tissue parts, and data from the SRA and CEO databases. Based on bioinformatics, expression analysis, and gene annotation results, the quinoa betalain biosynthesis genes *CYP76AD*, *DODA*, *B5GT*, and cDOPA 5-O-glucosyltransferase (c*DOPA*5GT) were cloned. The amount of betacyanins in green stems, red stems, red-striped stems, red leaves and green leaves was determined, and the relative expression of four genes in the three colors of quinoa’s green, red and red-striped stems, red and green leaves was studied. The results of this study will contribute to a better knowledge of the biosynthesis of quinoa betalains and the utilization of quinoa resources.

## 2. Materials and methods

### 2.1 Plant materials

As experimental material for gene cloning, eight quinoa germplasm resources with three seed colors, white 6, white 10, white 12, red 4, red 6, red 15, black 1 and black 3 were used (Figure S1). The betacyanin content and gene expression of betalain biosynthesis genes were determined based on the different colors revealed by the quinoa leaves and stems during the growth cycle, as well as five distinct leaves and stems: green stems, red stems, red-striped stems, red leaves, and green leaves. All samples were quickly frozen in liquid nitrogen and kept at a temperature of − 80°C until further usage, and all experiments were performed three times.

### 2.2 Gene identification and phylogenetic analyses

Based on documented betalain biosynthesis genes, betalain biosynthesis genes were downloaded from the NCBI database. Table S1 contains the GenBank accession numbers. Blast Zone, a TBtools package, was used to scan the quinoa genome for gene sequences.^42,43^ Gene sequences were carefully checked to remove duplicates and retain genes with more than or equal to 50% similarity. To ensure complete conserved domains, each candidate gene was further studied using the Conserved Domain Database (CDD). ExPasy (http://web.expasy.org/protparam/) was used to input the amino acid sequences of the acquired betalain biosynthesis genes *CqCYP76AD*, *CqDODA*, *CqGTs*,^44^ The chemical and physical features of the proteins encoded by the betalain biosynthesis genes *CqCYP76*, *CqDODA*, *CqGTs*, including amino acid length, molecular weight, and isoelectric point, were determined using the Compute pI/MW tool. The BUSCA website (http://busca.biocomp.unibo.it/) predicted the subcellular localization of the *CqCYP76AD*, *CqDODA*, and *CqGTs* genes.^45^

Multiple sequences were aligned using CLUSTAL (Bootstrap value = 1000). Protein evolutionary trees of quinoa *CqCYP76AD*, *CqDODA*, *CqGTs* and reported betalain biosynthesis genes were constructed by MEGAⅪ.^46^ The Neighbour Joining (NJ) method was used with the following parameters: the model was a ‘Poisson model’, the gap was set to ‘pairwise deletion’, the check parameter was bootstrap = 1000 times, and random seeds were used. The phylogenetic trees were then visualized using the iTOL online website (https://itol.embl.de/).^47^

### 2.3 Chromosomal distribution and gene duplication

TBtools v1 software was used to map the *CqCYP76AD*, *CqDODA* and *CqGTs* genes to quinoa chromosomes. The MCScanX multiple covariance scanning tool was used to analyze gene duplication events.^48^ TBtools v1 was used to visualize co-lineage maps of quinoa *CqCYP76AD*, *CqDODA*, and *CqGTs* genes. DnaSP6 was used to investigate the evolutionary selection connections of homologous genes within species.^49^

### 2.4 Analysis of gene structure characteristics and cis-acting elements

Quinoa genomic data were used to generate DNA and cDNA sequences of *CqCYP76AD*, *CqDODA* and *CqGTs*, which were then mapped using the online gene structure display system GSDS2.0 (http://gsds.cbi.pku.edu.cn/).^50^ MEME online program (http://meme-suite.org/) was used to analyze conserved domains, with the maximum number of conserved domains set to 10 motifs and the remaining set to default.^51^ The TBtools v1 was used to visualize the gene structure and conserved motif.^43^

The *CqCYP76AD*, *CqDODA*, *CqGT* promoter sequences (2000 bp) were extracted from the *C. quinoa* genome database, and cis-acting elements were predicted and analyzed using the PlantCARE online tool.^52^

### 2.5 Analysis of gene expression by the RNA-seq

Based on quinoa RNA-seq data under cold stress, quinoa tissue and seed germination expression data from CEO and SRA database, the tissue expression specificity of quinoa betalain biosynthesis genes *CqCYP76AD*, *CqDODA* and *CqGTs*, their role in seed germination and response to cold stress were investigated. The expression responses of *CqCYP76AD*, *CqDODA* and *CqGTs* gene members were studied in red and white quinoa under 4°C stress for 48 h. Based on transcriptome data of roots, stems, leaves, flowers, and fruits (accession numbers: GSE156523, GSE139174) from yellow quinoa, the tissue specificity of quinoa *CqCYP76AD*, *CqDODA*, *CqGTs* gene members was examined. During quinoa seed germination, the hypocotyl gradually changes color from white to red (Figure S2).^53^ The expression of *CqCYP76AD*, *CqDODA*, and *CqGTs* gene members was studied during seed germination to examine if hypocotyl color variations were related to betalain production. Therefore, the seed germination transcriptome data of red quinoa at 0 h,12 h,24 h,40 h,64 h were downloaded from the SRA database with the accession numbers SRR18766925, SRR18766924, SRR18766933, SRR18766932, SRR18766931, SRR18766930, SRR18766929, SRR18766928, SRR18766927, SRR18766926, SRR18766923, SRR18766922, SRR18766936, SRR18766935, SRR18766934. According to the quinoa genome database,^42^ mRNA abundance values were determined by the transcripts per million (TPM) method and transcript data (TPM) for *CqCYP76AD*, *CqDODA*, *CqGTs* gene members were heat mapped using TBtools v1.

### 2.6 Gene cloning and bioinformatics analyses

TransZol (TransGen Biotech, China) was used to extract total RNA from 3-leaf stage quinoa leaves. The TransScript® One-Step gDNA Removal and cDNA Synthesis SuperMix Reverse Transcription Kit (TransGen Biotech, China) was used to create the cDNA. AUR62028308, AUR62006953, AUR62004620, and AUR62013242 sequences were utilized as a reference to create specific primers based on the gene structure of *CqCYP76AD*, *CqDODA*, and *CqGTs* gene members, conserved structural domains, transcriptome expression correlation analysis, and gene annotation. Table S2 showed the primer sequences. The amplification products were eluted from the cDNA template according to the manufacturer’s instructions (TransGen Biotech, China), and the purified amplicons were coupled to the pEASY vector. After transforming Trans-T1 *Escherichia coli* into a receptor state, positive single colonies were selected, grown, and delivered to Shanghai Biotech for sequencing.

TMHMM (http://www.cbs.dtu.dk/services/TMHMM/) and Protscale (https://web.expasy.org/protscale/) were used to predict protein transmembrane structural domains and hydrophobicity. The phosphorylation sites of the protein were predicted using NetNGly 1.0 (http://www.cbs.dtu. dk/services/NetNGlyc/). The secondary and three-dimensional structures of the protein were predicted using SOPMA with SWISS-MODEL.

### 2.7 Analysis of the betalain content and gene expression of betalain

Five quinoa stems and leaves with significantly different colors were taken to investigate the relationship between the content of betacyanins and the expression of *CqCYP76AD*, *CqDODA*, and *CqGTs* genes in the stems and leaves of different traits of quinoa: green stems, red stems, red-striped stems, red leaves, and green leaves (Figure S1). The betacyanin content was calculated using the Nemzer technique for extracting betacyanin,^54^ as shown below, Bp: betalain content (mg/g), A: absorbance (538 nm), DF: dilution multiple, Mω: relative molar mass of betacyanin (550 g/mol), V: volume (L), ε: betacyanin extinction coefficient (61600 L/mol·cn), L: length of light range (1 cm), m: mass (g). Correlation analysis between betacyanin content and betalain biosynthesis genes expression was conducted using the Origin software.

$$Bp=\frac{A\times DF\times M\omega \times 1000\times V}{\varepsilon \times L\times m}$$

Real-time fluorescence quantitative primers were designed by the cloned sequences of the *CqCYP76AD*, *CqDODA*, *CqGTs* genes, and the primer sequences were listed in Table S2. Total RNA was extracted from green stems, red stems, red-striped stems, red leaves and green leaves using a total plant RNA extraction kit (TransGen Biotech, China), and complementary DNA was synthesized using reverse transcriptase HiScript III RT superMix (R333, vazyme). Then qRT-PCR was performed with Vazyme SYBR premixed Taq Pro Universal (Q712,vazyme), and gene expression was calculated using the 2-^∆∆CT^ method.^54^ All data analysis was performed in Excel 2019, analysis of variance was performed in SPSS v26, and the least significant difference (LSD) multiple comparison method was used.

## 3. Results

### 3.1 Identification and classification of betalain biosynthesis genes

A genome-wide screening of quinoa was performed using TBtools v1 and was based on previously identified sequences of genes associated to betalain production in the order Caryophyllales. These potential genes were then manually evaluated for the completeness of their conserved structural domains. In the quinoa genome, 59 potential genes for betalain production were found, including 17 *CqCYP76AD* genes, 12 *CqDODA* genes, and 30 *CqGTs* genes (Table S3). The physicochemical analysis of the three betalain biosynthesis genes revealed that all the *CqCYP76AD* genes encoded proteins were hydrophilic, with only four proteins being unstable, and that only AUR62022993 was distributed in the organelle membrane, while the rest were distributed in the inner membrane system. The isoelectric points of the proteins expressed by the *Cq*DODA gene were all less than 7. Five of the proteins were unstable hydrophilic proteins, while the remaining seven were stable hydrophilic proteins. AUR62006955 was found in the chloroplast, AUR62006951 and AUR62006956 in the nucleus, and the remainder in the cytoplasm. The isoelectric points of all the proteins encoded by the GTs gene were less than 7. Seven proteins were unstable hydrophilic proteins and the rest were stable hydrophilic proteins. Only AUR62022641 was found in the chloroplasts while the others were found in the plasma membrane. The quinoa betalain biosynthesis gene proteins’ physicochemical characteristics and subcellular localization were compatible with the conditions necessary for the betalain biosynthesis gene to function.

**Figure 2.**
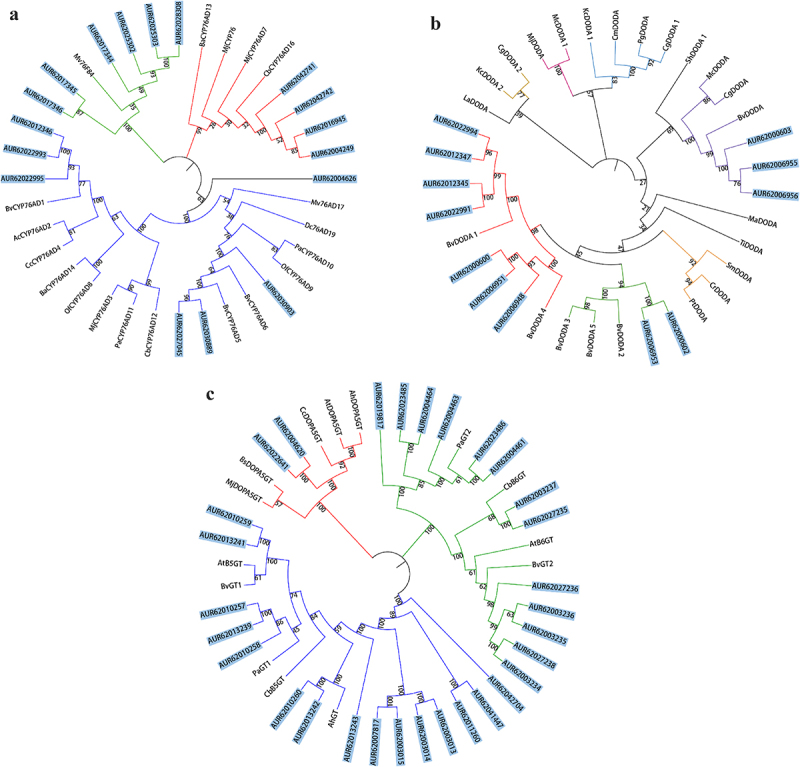
Phylogenetic analysis of *CqCYP76AD* (a), *CqDODA* (b) and *CqGts* (c) with the other plant species. (a) the blue line indicates α evolutionary branch, the green line indicates β evolutionary branch and the red line indicates γ evolutionary branch. (b) the purple line indicates α evolutionary branch, the others belong to β evolutionary branch. (c) the red line indicates *Cq*c*DOPA5GT* evolutionary branch, the blue line indicates *CqB5GT* evolutionary branch and the green line indicates *CqB6GT* evolutionary branch. Blue label background indicates genes from *C. quinoa*.

Three phylogenetic trees were created with known genes relevant to betalain biosynthesis in Caryophyllales to analyze the evolutionary connections of the 59 candidate genes (Figure 2）. The 17 *Cq*CYP76AD proteins were divided into three branches, CYP76AD-α, CYP76AD-β and CYP76AD1-γ. The CYP76AD-α branch had 7 members and synthesized L-DOPA and cycloDOPA. The CYP76AD-β branch had 6 members and had tyrosine hydroxylase activity. There were 4 members on the CYP76AD1-γ branch and the function of the CYP76AD1-γ branch was unknown. According to the phylogenetic tree 12 *Cq*DODA proteins were divided into 9 DODAα and 3 DODAβ. In the *Cq*GTs phylogenetic tree, GTs were divided into three groups, B5GT, B6GT and cDOPA5GT. Among the 30 GTs proteins, 15 belonged to B5GT, 13 to B6GT and 2 to cDOPA5GT.

### 3.2 Chromosomal localization and replication of quinoa betalain biosynthesis genes

**Figure 3.**
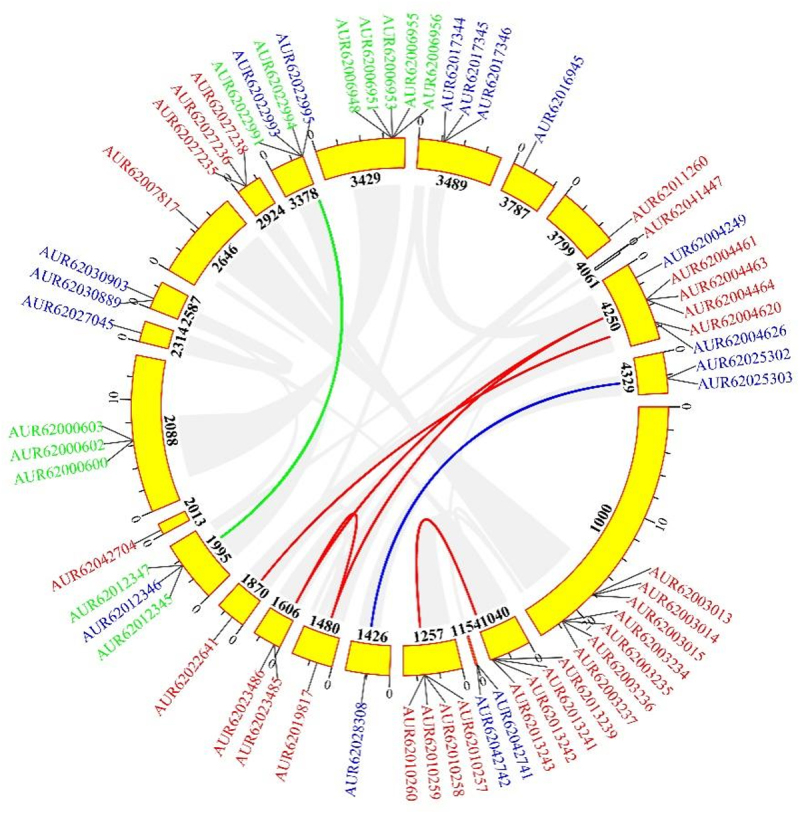
Distribution and duplication of segments of *CqCYP76AD*, *CqDODA* and *CqGts* genes in *C. quinoa*.

The co-linear regions were studied using the MCscanX software to provide light on the replication of betalains biosynthesis genes in quinoa. This study found 8 duplicated gene pairs, including 2 duplicated gene pairs for *CqCYP76AD*, 1 duplicated gene pair each for *CqB5GT*, *CqB6GT*, *Cq*c*DOPA5GT*, and *CqDODA*, and 2 duplicated gene pairs between *CqB5GT* and *CqB6GT* (Figure 3). *CqCYP76AD* possessed two duplicated genes at a chromosomal position comparable to *CqDODA*, implying that *CqCYP76AD* was evolutionarily linked to *CqDODA*. To analyze the molecular evolutionary rates of quinoa betalain biosynthesis genes, non-synonymous substitutions (Ka), synonymous substitutions (Ks) and Ka/Ks ratios amongst covariate genes were calculated. The Ka/Ks ratios of the duplicated genes were all smaller than one, showing that the quinoa betalain biosynthesis co-linear genes were under to purifying selection during evolution (Table 1).

**Table 1.** Ka/Ks analysis of duplicate gene pairs of *CqCYP76AD*, *CqDODA* and *CqGts* in *C. quinoa*..

Duplicated gene1	Duplicated gene2	Subfamily	Ka	Ks	Ka/Ks	Purifing Selection
AUR62013239	AUR62010257	B5GT	0.02	0.08	0.29	Yes
AUR62028308	AUR62025302	CYP76AD	0.15	0.63	0.24	Yes
AUR62019817	AUR62023485	B5GT/B6GT	0.42	2.09	0.20	Yes
AUR62019817	AUR62004461	B5GT/B6GT	0.44	1.67	0.26	Yes
AUR62023485	AUR62004461	B6GT	0.37	2.17	0.17	Yes
AUR62022641	AUR62004620	cDOPA5GT	0.02	0.14	0.17	Yes
AUR62012345	AUR62022991	DODA	0.02	0.11	0.23	Yes
AUR62012346	AUR62022993	CYP76AD	0.01	0.14	0.06	Yes

### 3.3 Analysis of gene characteristics and cis-acting elements

**Figure 4.**
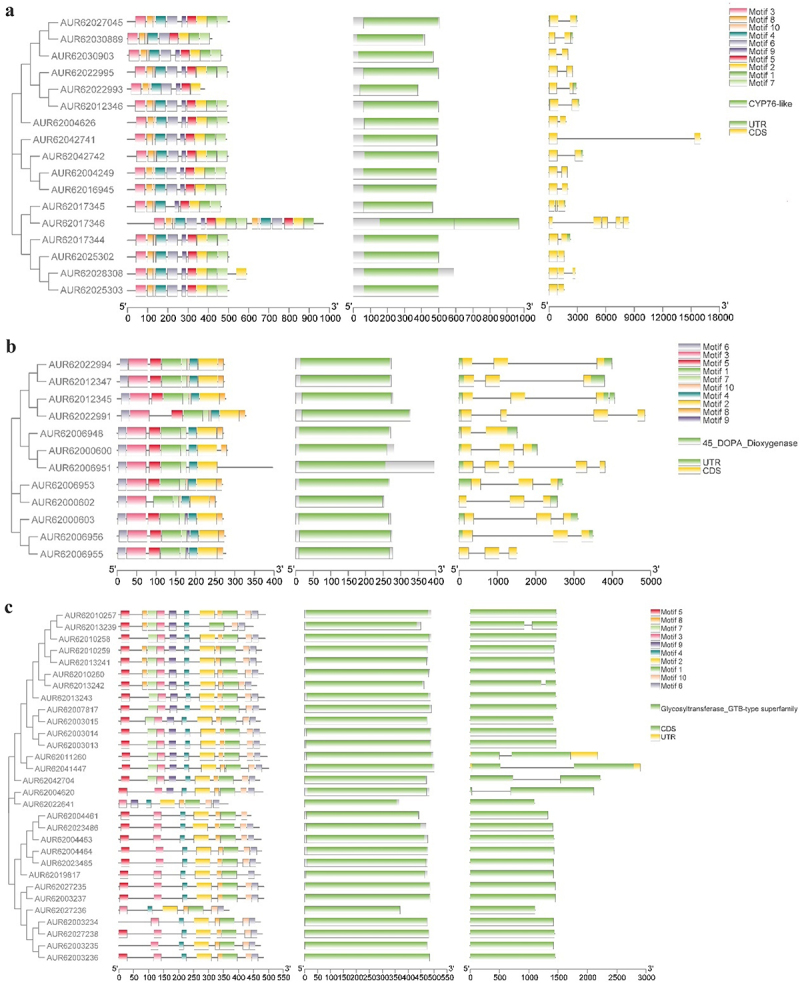
Conserved motifs, conserved structural domains and gene structures of *CqCYP76AD*, *CqDODA* and *CqGts* genes based on phylogenetic relationships in *C. quinoa*. (a) *CqCYP76AD* gene characterization. (b) *CqDODA* gene characterization. (c) *CqGts* gene characterization.

A total of 10 different motifs, conserved domains, and gene structures were identified to analyze the structural features of the quinoa betalain biosynthesis genes using the MEME motif search tool, the Conserved Domain Database (CDD), and the online gene structure display system GSDS2.0, and the three were finally integrated by TBtools v1 (Figure 4). Except for AUR62030889, AUR62022993, AUR62017345, AUR62017346 and AUR62028308, all the *CqCYP76AD* gene members made up the 10 dispersed, quantitatively consistent Motif conserved structural domains identified in the investigation. Except for AUR62017345, AUR62017346 and AUR62028308, all 14 members had two exons. AUR62042741 was more than 15,000 bp long, but it also had just two exons and shared structural domains with other *CqCYP76AD* genes. When combined with the conserved structural domain and conserved motif, the gene AUR62017346 contained two CYP76-like conserved structural domains and seven more repeat motifs than the other genes. The six conserved motifs Motif1, Motif2, Motif3, Motif4, Motif6, and Motif7 were used by all *CqDODA* gene members, and *CqDODA* was classified into four branches based on the Motifs they included. The *CqDODA* gene members had three exons, except AUR62006948, which had two exons, AUR62022991, which had four exons, and AUR62006951, which had five exons. The variance in intron length of each gene resulted in varied gene lengths, although the difference in coding region length was not significant. Motif1,3,4,6 was shared by the *CqB5GT*, *CqB6GT* and *Cq*c*DOPA5GT* genes among *CqGTs* members. The conserved motifs included by *CqB5GT*, *CqB6GT* and *Cq*c*DOPA5GT* were separated into three branches, with Motif7 including just *CqB5GT* and Motif9 which included both *CqB5GT* and *Cq*c*DOPA5GT*. In terms of gene structure, *CqB5GT* contained five genes, whereas *Cq*c*DOPA5GT* had one gene with an intron. Only the four genes with introns had longer gene lengths, while the rest of the genes were not significantly different. Although there was no significant difference in gene structure between *CqB5GT* and *CqB6GT*, the sequence and function of CqB5GT and CqB6GT proteins were different, and more research on the structure and function of CqB5GT and CqB6GT proteins is required.

**Figure 5.**
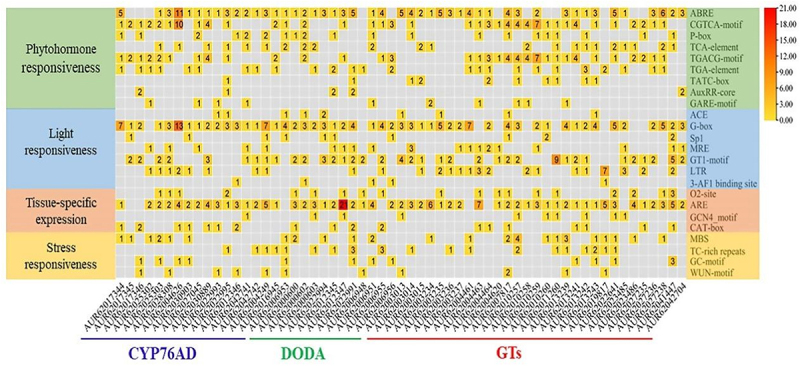
Analysis of the cis-acting elements of the *CqCYP76AD*, *CqDODA* and *CqGts* genes.

Transcription factors that affect gene expression are known as cis-acting regulatory elements. To investigate further the biological functions that quinoa betalain biosynthesis genes may be involved in, 59 quinoa betalain biosynthesis genes were predicted for upstream promoter elements using PlantCARE, and the number of endogenous hormone, tissue-specific expression, stress, and light response-related cis-acting elements were examined (Figure 5). Endogenous hormones, light response, tissue-specific expression, and stress all had at least one cis-acting element. Endogenous hormone cis-acting elements reacting to growth hormone, gibberellin, salicylic acid, abscisic acid, and methyl jasmonate were more frequent. The cis-acting elements in response to adversity are mainly associated with cold and drought stress.

### 3.4 Analysis of expression patterns of quinoa betalain biosynthesis genes

**Figure 6.**
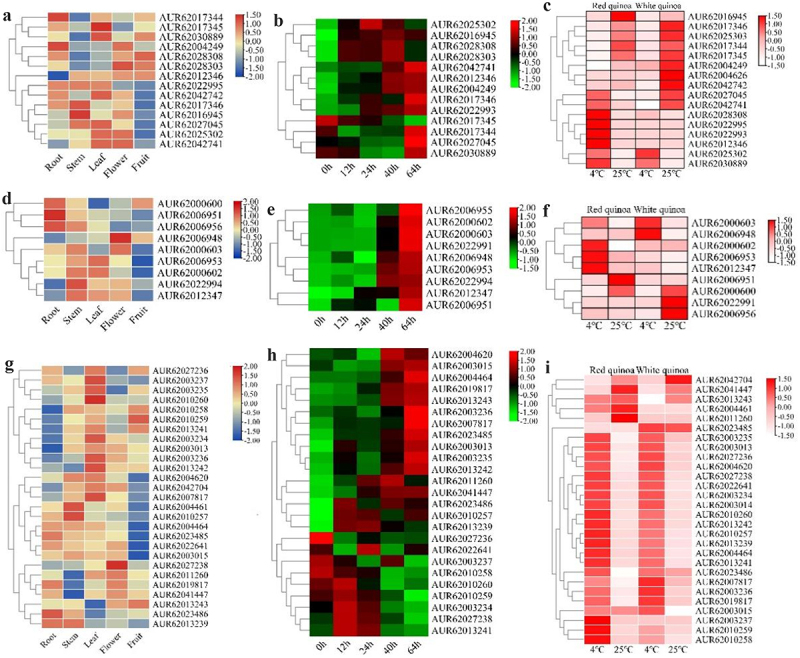
Heat map of quinoa betalain synthesis gene expression. (a, b, c) expression of *CqCYP76AD* gene members in different tissues, seed germination and cold stress. (d, e, f) expression of *CqDODA* gene members in different tissues, seed germination and cold stress. (g, h, i) expression of *CqGts* gene members in different tissues, seed germination and cold stress.

To predict their functions in different tissues, seed germination, and cold stress, the expression patterns of *CqCYP76AD*, *CqDODA* and *CqGTs* genes associated to quinoa betalain production were examined in distinct tissues, seed germination, and cold stress. A heat map was used to show the expression pattern of quinoa betalain biosynthesis genes (Figure 6). As shown in the graph, the number of genes expressed by each member of the *CqCYP76AD*, *CqDODA*, *CqGTs* was much higher in stems, leaves, and flowers than in roots and fruits, with the maximum number of genes strongly expressed in leaves and the lowest in fruits. *CqCYP76AD*, *CqDODA* and *CqGTs* were all found in high level in roots, stems, leaves, and flowers, but only four *CqB5GT* genes, AUR62013243, AUR62010259, AUR62013241 and AUR62010258, and one *CqCYP76AD* gene, AUR62028308, were found in high level in fruits, probably as fruit-specific genes. Except for fruits, both *CqcDOPA5GT* genes were expressed in roots, stems, leaves, and flowers. As seeds germinated, betalains accumulated in the hypocotyl (Figure S2), and the expression of most genes involved in betalain biosynthesis gradually increased with seed germination, with all *CqDODA* gene members showing this trend. Hypocotyl betalains had not accumulated before 40 hours of seed germination, but some of the *CqCYP76AD*, *CqGTs* genes were strongly expressed, indicating that *CqCYP76AD*, *CqGTs* genes were also engaged in other physiological functions in quinoa. The expression of genes involved in betalain biosynthesis was compared in red and white quinoa seedlings at the 5-leaf stage under cold stress to see if betalains were involved in cold stress. After 48 h of stress at 4°C, *CqCYP76AD*, *CqDODA* and *CqGTs* were significantly expressed compared with the control, quinoa betalain is thought to be involved in cold stress.

### 3.5 Gene cloning and bioinformatics analysis

The sequences of *CqCYP76AD*, *CqDODA*, *Cq*c*DOPA5GT*, and *CqB5GT* genes were acquired by RT-PCR from three red quinoas, three white quinoas, and two black quinoas to determine if there were variations in the structure of genes associated to the varied colors of quinoa betalain biosynthesis (Figure S3). The gene fragment size was as predicted, and sequencing analysis revealed that the inserts were *CqCYP76AD*, *CqDODA*, *Cq*c*DOPA5GT* and *CqB5GT* gene CDS, which may be used for future investigation. The nucleotide sequences of the *CqCYP76AD*, *CqDODA*, *Cq*c*DOPA5GT* and *CqB5GT* genes in the eight quinoa samples were 100% identical, as were the gene structures.

**Figure 7.**
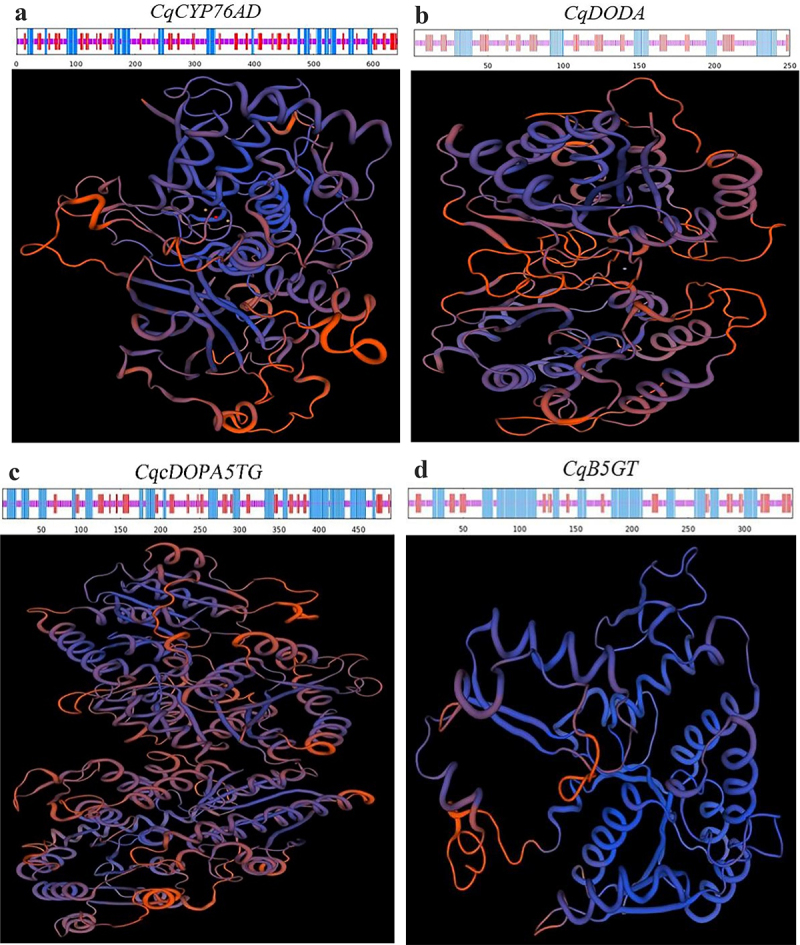
Secondary and tertiary structure of the quinoa betalain synthesis genes. (a) Secondary and tertiary structure of the C*qCYP76AD* gene in *C.Quinoa*. (b) Secondary and tertiary structure of the C*qDODA* gene in *C.Quinoa*. (c) Secondary and tertiary structure of the C*qcDOPA5GT* gene in *C.Quinoa*. (d) Secondary and tertiary structure of the C*qB5GT* gene in *C.Quinoa*.

Further bioinformatics investigation of the four genes *CqCYP76AD*, *CqDODA*, *CqcDOPA5GT* and *CqB5GT* in quinoa revealed that all four genes were unstable hydrophilic proteins (Figure S4). Protein phosphorylation site predictions revealed that serine phosphorylation was prevalent in the phosphorylation of four genes, indicating that serine phosphorylation plays a major role in the activity of quinoa betalain production genes. The CqCYP76AD protein was made up of 22.46% alpha-helix, 18.88% extended chain, and 58.66% random curl. CqDODA contained 21.2% alpha-helix, 22.4% extended chain, and 56.4% random curl. CqcDOPA5GT was made up of 35.44% alpha-helix, 17.92% extended chain, and 46.64% random curl. CqB5GT had 39.94% alpha-helix, 17.78% extended chain, and 42.27% random curl (Figure 7).

### 3.6 Determination of betacyanin contents

**Figure 8.**
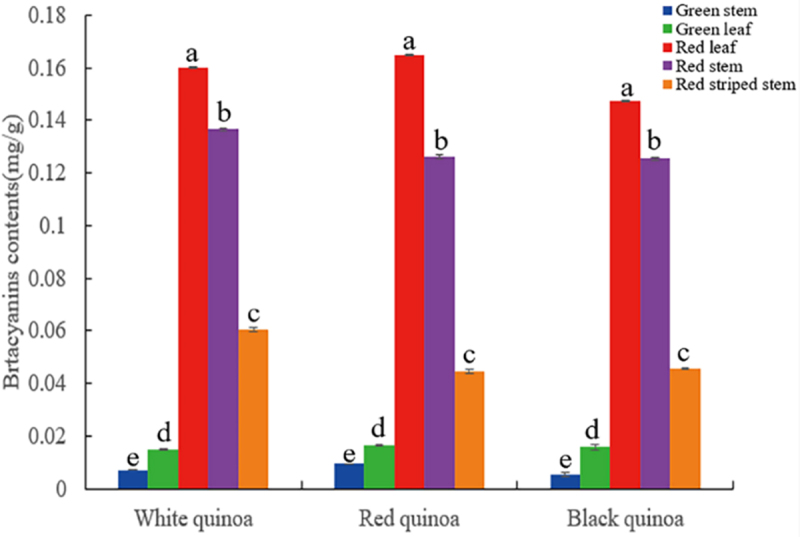
Analysis of betacyanin contents in stems and leaves of different phenotypes of *C. quinoa*.

The betacyanin contents of green stems, red stems, red-striped stems, red leaves and green leaves of white, red and black quinoa were measured to analyze the betacyanin accumulation of different colors of quinoa. The results revealed that the betacyanin contents of the stems and leaves of the five different traits differed significantly, with the betacyanin contents of red leaves, red stems, red-striped stems, green leaves and green stems decreasing in order, while there were no significant differences in the betacyanin contents of the different colors of quinoa (Figure 8).

### 3.7 Expression analysis of genes related to betalain biosynthesis in stems and leaves of different characters

**Figure 9.**
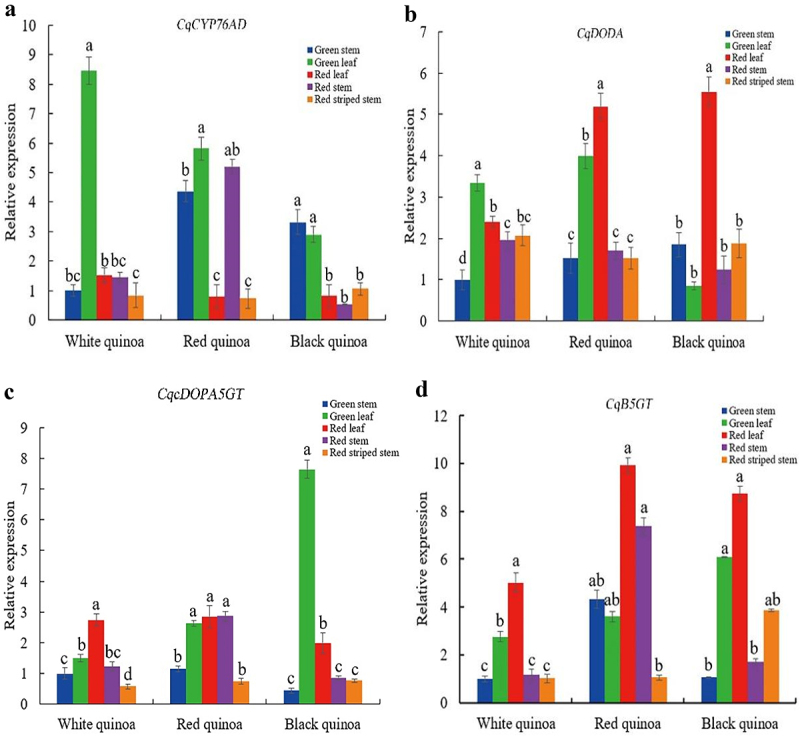
Expression patterns of quinoa betalain synthesis genes in stems and leaves of different traits. (a) expression pattern of the *CqCYP76AD* gene in different coloured quinoa stems and leaves. (b) expression pattern of the *CqDODA* gene in different coloured quinoa stems and leaves. (c) expression pattern of the *CqcDOPA5GT* gene in different coloured quinoa stems and leaves. (d) expression pattern of the *CqB5GT* gene in different coloured quinoa stems and leaves. Different lowercase letters indicate significant differences at *P* < 0.05.

Based on the findings of the betacyanin contents, the expression quantities of genes involved in betalain production in green stems, red stems, red-striped stems, red leaves and green leaves of white, red and black quinoa were investigated (Figure 9). The expression of *CqCYP76AD* gene was significantly higher in green leaves than in red leaves, red stems, red-striped stems and green stems of white quinoa. In red quinoa, the expression in green leaves was significantly higher than those in red leaves, red-striped stems and green stems, while the expression between green stems and red stems was not significant, but significantly higher than those in red leaves and red-striped stems. Expression in green leaves and green stems was significantly higher than those in red leaves, red stems and red striped stems of black quinoa. The *CqCYP76AD* gene showed significantly higher expression in green leaves than in other traits of stems and leaves in white, red and black quinoa. The expression of *CqDODA* gene was significantly higher in green leaves than in red leaves, red stems, red-striped stems and green stems of white quinoa, which was consistent with the trend of *CqCYP76AD* gene expression. The expression in red leaves of red and black quinoa was significantly higher than those in other trait stems and leaves, while the expression in red stems, red-striped stems, and green stems was not significantly different. The *CqcDOPA5GT* gene was significantly higher expressed in the red leaves of white quinoa than in the green leaves, red stems, red-striped stems and green stems. In red quinoa, the expression was not significantly higher in green leaves, red leaves and red stems, but significantly higher than those in green stems and red-striped stems. In black quinoa the expression was significantly higher in green leaves than in the other trait stems and leaves, and significantly higher in red leaves than in red stems, red-striped stems and green stems. The *CqcDOPA5GT* gene was the least expressed in the red-striped stems of white, red and black quinoa. The *CqB5GT* gene was significantly more expressed in the red leaves of white quinoa than in the other traits stems and leaves, which was consistent with the trend of *CqDOPA5GT* gene expression. In red quinoa expression was not significant among green stems, red stems, red leaves and green leaves, but significantly higher than in red-striped stems. The expression was significantly higher in red leaves and green leaves than in green stems and red stems of black quinoa. Correlation study of gene expression and betacyanin contents showed no significant relationship between the expression of quinoa betalain biosynthetic genes and betacyanin contents (Figure S5).

## 4. Discussion

Quinoa is gaining popularity because to its high nutritional content and adaptability to a variety of environments.^40,55^ Because of its natural drought and salt resistance, quinoa has become a popular crop for barren, saline and dry areas.^56^ Quinoa fruit is excellent in nutrition, with a high protein content, a balanced amino acid ratio, and a high level of dietary fiber, mineral elements, and vitamins. It is appropriate for a wide range of people, particularly pregnant women, celiac disease sufferers, and diabetics.^55,57^ Natural betalains is now obtained mostly from beetroot, and the need for betalains on the international market increases every year.^58,59^ The presence of betalains enables quinoa to seem colorful, and the mature quinoa fruit is so colorful that 66 different colors of quinoa seeds have been observed in Bolivia’s quinoa harvesting area.^38,39^ The betalains found in red and black quinoa fruits are primarily betanin and isobetanin, while the betalains found in red-purple fruits are primarily amaranthin and isoamaranthin, and the pigments found in yellow quinoa fruits are dophanin, dopamine betaxanthin and vulgaxanthin of the betaxanthin family.^38,39^ Quinoa’s various betalains may be the next natural source of betalains as a way to improve the utilization of quinoa stems and leaves. Quinoa morphology differs significantly between variations, and the same variety exhibits distinct features at different development stages, with leaves mostly displaying red, green, and yellow stripes and stems displaying red, green, and red stripes. As a result, we attempted to investigate the potential roles by examining the structural properties, tissue expression specificity, and expression patterns of genes involved in the production of quinoa betalains during seed germination and cold stress. The relative expression of *CqCYP76AD*, *CqDODA*, *Cq*c*DOPA5GT* and *CqB5GT* in quinoa green stems, red stems, red-striped stems, red leaves and green leaves was investigated by cloning the four genes and extracting the content of betalains in green stems, red stems, red-striped stems, red leaves and green leaves, in order to investigate the relationship between the structure, expression, and betacyanin contents of the four genes of *C. quinoa*.

We discovered 59 quinoa betalain biosynthesis genes and performed gene structure characterization, tissue expression specificity, and expression analysis during seed germination and cold stress. Cloning yielded *CqCYP76AD*, *CqDODA*, *Cq*c*DOPA5GT* and *CqB5GT* genes, and the link between gene expression and betacyanin contents was investigated. The 59 genes identified were divided into evolutionary trees with known functions of betalain biosynthesis genes to study the evolutionary relationships of genes related to quinoa betalain biosynthesis. According to available studies,^60,61^ *CqCYP76AD* genes were classified as ɑ, β and γ types, *CqDODA* as ɑ and β types, and *GTs* as *Cq*c*DOPA5GT*, *CqB5GT* and *CqB6GT*. The covariance findings revealed eight duplicated gene pairs in 59 genes, including two pairs of duplicated genes between *CqB5GT* and *CqB6GT*. In terms of chromosomal location, one set of duplicated genes in *CqCYP76AD* was comparable to the duplicated genes in *CqDODA*, therefore it is assumed that *CqCYP76AD* is evolutionarily linked to *CqDODA*. The Ka/Ks ratios of the duplicated genes were all smaller than one, showing that purifying selection acted on the quinoa betalain biosynthesis co-linear genes during the evolutionary process. The structural properties of the genes demonstrate that all three categories of genes, *CqCYP76AD*, *CqDODA* and *CqGTs*, had associated conserved structural domains, and the number and distribution of Motifs may differ across the different types of genes.

The analysis of promoter cis-acting elements revealed that *CqCYP76AD*, *CqDODA*, *CqGTs* had cis-acting elements linked with endogenous hormones, tissue-specific expression, stress, and light response, which was consistent with earlier studies.^62^ The highest number of cis-acting elements reacting to endogenous hormones, followed by light-responsive cis-acting elements, is thought to be related to antioxidant ability of betalains and their regulator MYB.^63,64^ Visual inspection revealed that quinoa stems and leaves were high in betalains, and tissue-specific investigation of *CqCYP76AD*, *CqDODA* and *Cq*GTs revealed that the number of genes highly expressed in stems and leaves was substantially greater than in roots and fruits. During quinoa seed germination, betalains gradually accumulated in the hypocotyl, resulting in elevated expression of genes involved in betalain production.^53^ At 64 hours of seed germination, all *CqDODA* genes were highly expressed, as were the majority of *CqCYP76AD* and *CqGTs* genes, indicating that *CqDODA* genes play an important role in the pre-synthesis stage of betalain biosynthesis. This is because only *CqDODA* genes are involved in the betalain production pathway, where they manufacture the betalain precursor, betalains acid.^29,61^ Quinoa is grown in most highland places, and it is acclimated to cold, thus we wanted to see if quinoa betalains have a role in cold resistance. *CqCYP76AD*, *CqDODA* and *CqGTs* genes were considerably upregulated in red and white quinoa after 48 h of stress at 4°C compared to the control, quinoa betalain is thought to be involved in cold stress.

The sequences of *CqCYP76AD*, *CqDODA*, *Cq*c*DOPA5GT*, *CqB5GT* genes were cloned from three red quinoas, three white quinoas, and two black quinoas to see if there were changes in the sequences of genes associated to betalain production in various colors of quinoa. The nucleic acid sequences of the *CqCYP76AD*, *CqDODA*, *Cq*c*DOPA5GT* and *CqB5GT* genes were found to be identical. Bioinformatics investigation of the proteins encoded by the four genes indicated that they are all unstable hydrophilic proteins with alpha-helix, extended chain, and random curl. *CqCYP76AD* and *CqDODA*, *CqcDOPA5GT* and *CqB5GT* all had comparable proportions of alpha-helix, extended chain, and random curl. It was suggested that *CqCYP76AD* and *CqDODA*, *CqcDOPA5GT* and *CqB5GT* would be evolutionarily connected in genetic inheritance when combined with gene duplication and chromosomal position. The content of betacyanin and the expression of the *CqCYP76AD*, *CqDODA*, *CqcDOPA5GT* and *CqB5GT* genes in the stems and leaves of various quinoas revealed that the betacyanin content of the red stems and leaves was significantly higher than that of the green stems and leaves, which was consistent with visual observations. However, the levels of expression of *CqCYP76AD*, *CqDODA*, *CqcDOPA5GT* and *CqB5GT* genes in different stems and leaves had no obvious relationship with betacyanin contents, which is consistent with the results of related experiments in amaranth.^65^ B5GT and B6GT are involved in the glycosylation of flavonoids, and previous study showed that post-transcriptional gene silencing regulates betalain biosynthesis.^36,66^ This might explain why variations in their expression levels do not always correlate with betacyanin contents.

## 5. Conclusion

The 59 *CqCYP76AD*, *CqDODA*, *CqGTs* betalain biosynthesis genes were found in quinoa, and the findings of gene structure, gene placement, and gene duplication showed that *CqCYP76AD* and *CqDODA*, *Cq*c*DOPA5GT* and *CqB5GT* may have an evolutionary linkage genetic influence. RNA-seq data analysis revealed that the genes *CqCYP76AD*, *CqDODA* and *CqGTs* were highly expressed in tissue regions where betalains were deposited. With the elongation of the hypocotyl during seed germination, betalains were rapidly synthesized. The hypocotyl became red at 64 hours of germination, and all members of the *CqDODA* gene, as well as most members of the *CqCYP76AD* and *Cq*GTs genes, were strongly expressed, showing that the *CqDODA* gene is critical in the early phases of betalain production. *CqCYP76AD*, *CqDODA* and *CqGTs* genes were considerably upregulated after 48 h of stress at 4°C compared to the control, and it is assumed that quinoa betalains are involved in cold stress. The *CqCYP76AD*, *CqDODA*, *Cq*c*DOPA5GT*, *CqB5GT* gene sequences were similar across eight distinct quinoa colors, demonstrating that the *CqCYP76AD*, *CqDODA*, *CqcDOPA5GT*, *CqB5GT* genes are highly conserved in quinoa. Furthermore, the inconsistency between changes in gene expression and betalain accumulation indicates that other factors may influence betalain biosynthesis in quinoa. In conclusion, the quinoa *CqCYP76AD*, *CqDODA*, *CqGTs* genes are important in betalain biosynthesis. This research establishes the groundwork for understanding the molecular mechanisms underlying quinoa color variability and gives guidelines for the full usage of betalains in quinoa plants.

## Supplementary Material

Supplemental Material
