# Supplementary figures and images for: Identification, expression analysis of quinoa betalain biosynthesis genes and their role in seed germination and cold stress
Source: Plant Signal Behav. 2023 Aug 24;18(1):2250891. doi: 10.1080/15592324.2023.2250891 (PMC10453985; doi:10.1080/15592324.2023.2250891)

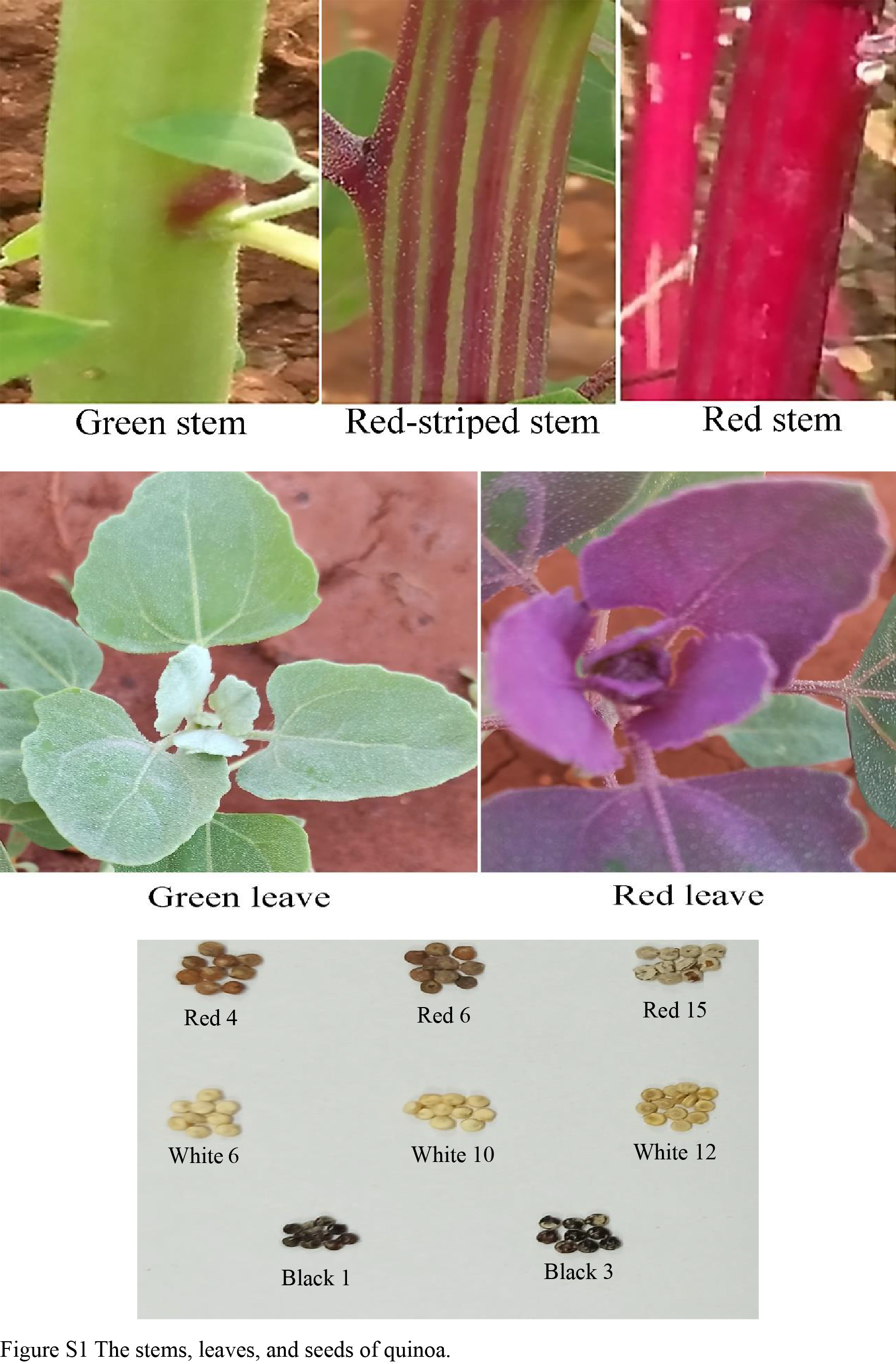

Supplement: Supplemental Material [file KPSB_A_2250891_SM9138.zip › Figure S1.png]

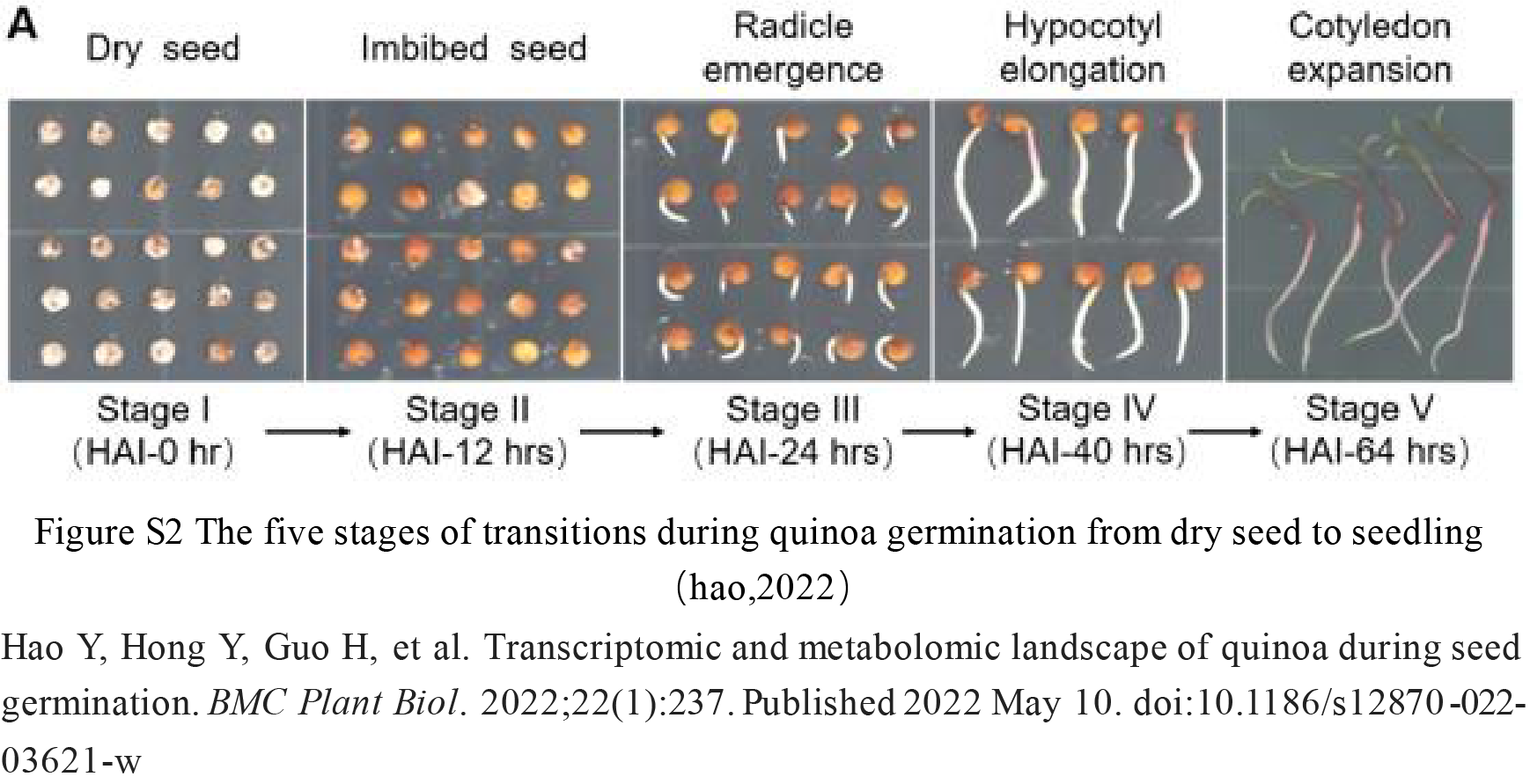

Supplement: Supplemental Material [file KPSB_A_2250891_SM9138.zip › Figure S2.png]

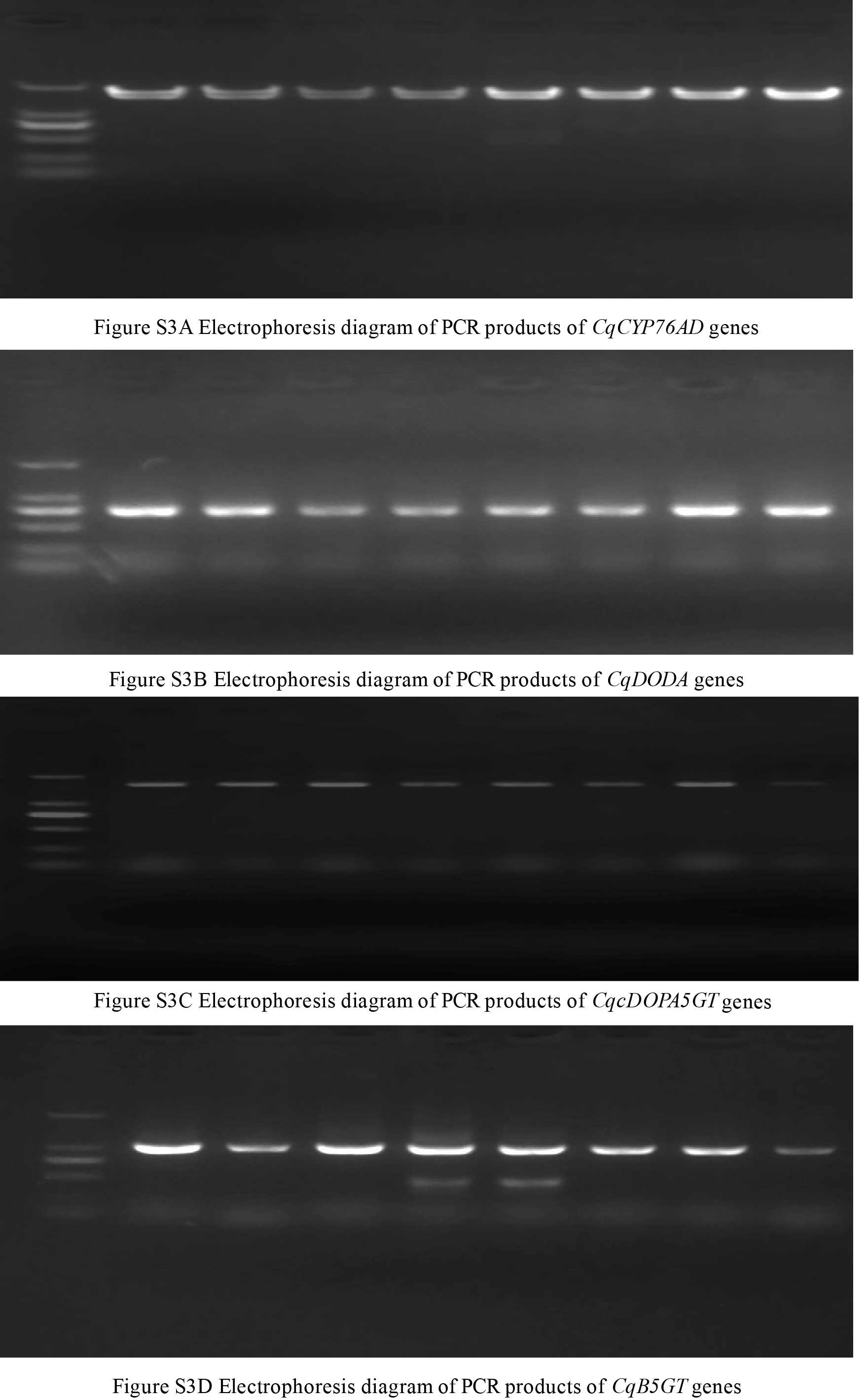

Supplement: Supplemental Material [file KPSB_A_2250891_SM9138.zip › Figure S3.png]

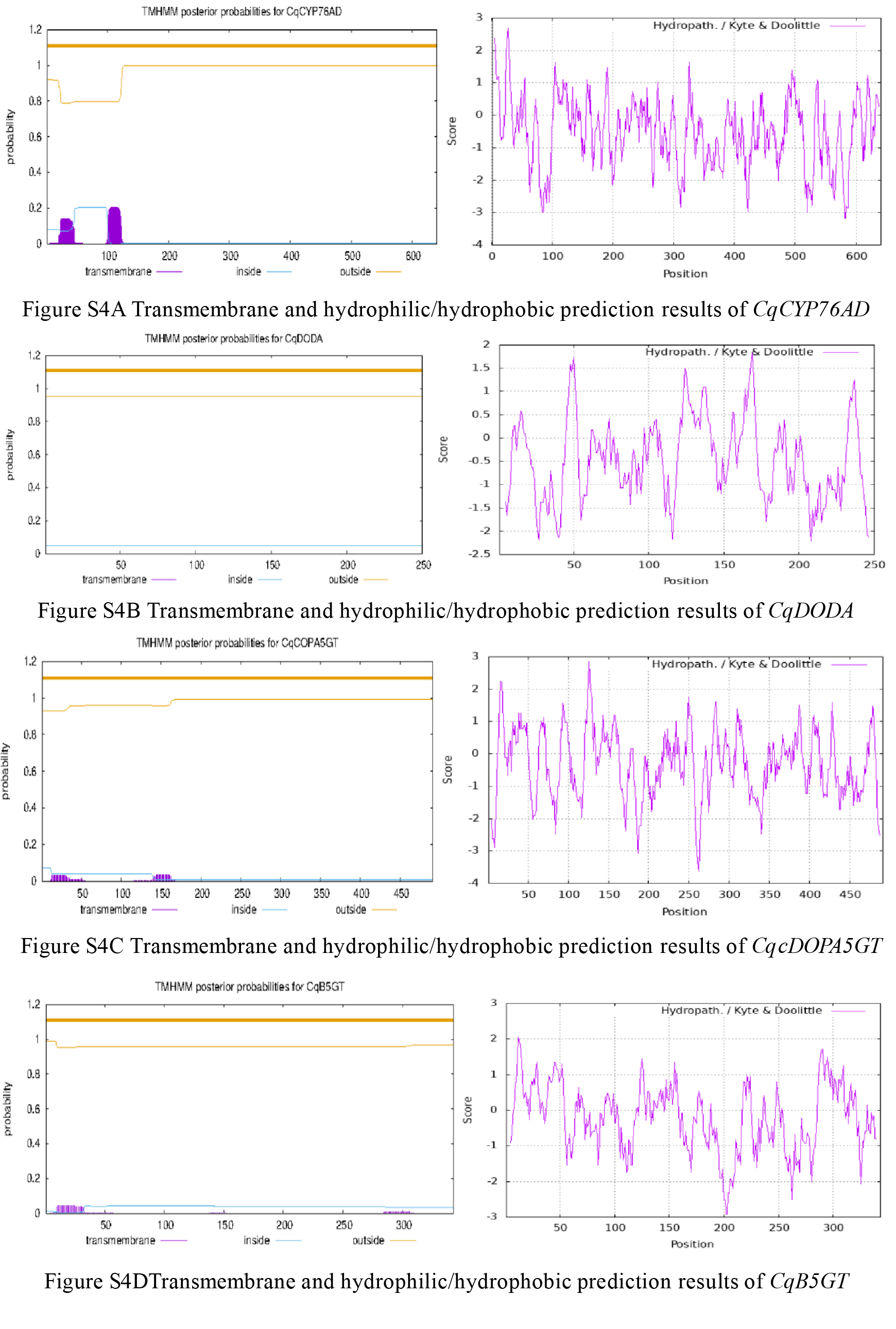

Supplement: Supplemental Material [file KPSB_A_2250891_SM9138.zip › Figure S4.png]

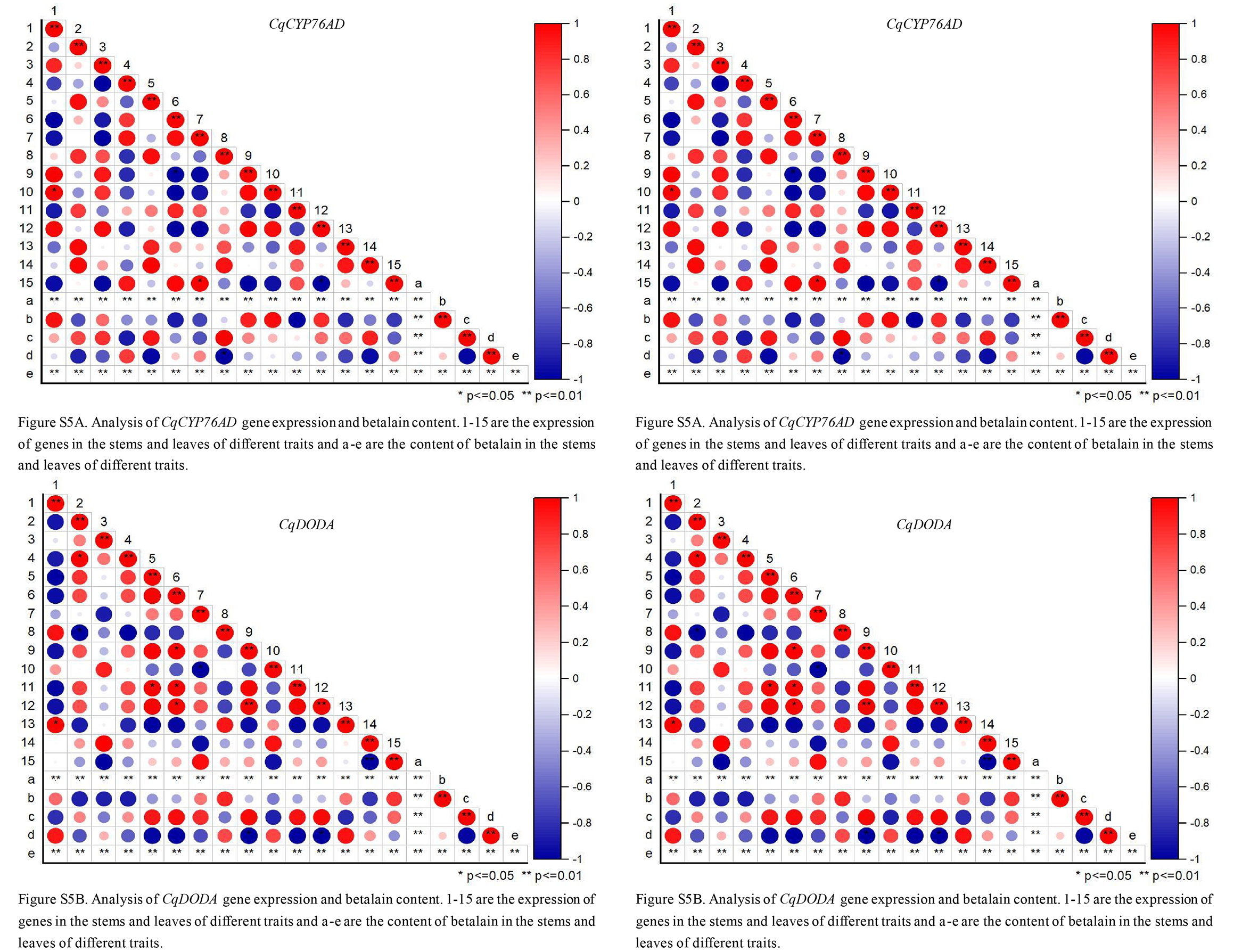

Supplement: Supplemental Material [file KPSB_A_2250891_SM9138.zip › Figure S5.png]
